# Alterations of adipokines, pancreatic hormones and incretins in acute and convalescent COVID-19 children

**DOI:** 10.1186/s12887-023-03971-w

**Published:** 2023-04-03

**Authors:** Anuradha Rajamanickam, Aishwarya Venkataraman, Nathella Pavan Kumar, R. Sasidaran, Arul Nancy Pandiarajan, Nandhini Selvaraj, Ruchi Mittal, K. Gowshika, Sulochana Putlibai, S. Lakshan Raj, Padmasani Venkat Ramanan, Subash Babu

**Affiliations:** 1 National Institutes of Health-National Institute for Research in Tuberculosis - International Center for Excellence in Research, Chennai, India; 2 ICMR-National Institute for Research in Tuberculosis, Chennai, India; 3 Kanchi Kamakoti CHILDS Trust Hospital, Chennai, India; 4 Sri Ramachandra Institute of Higher Education & Research, Chennai, India; 5 Laboratory of Parasitic Diseases, National Institute of Allergy and Infectious Diseases, National Institutes of Health, Bethesda, MD USA

**Keywords:** Adipokines, Pancreatic hormones and Incretins, Acute COVID-19 children, Convalescent COVID-19 children

## Abstract

**Background:**

The Severe Acute Respiratory Syndrome Coronavirus-2 (SARS-CoV-2), accountable for Coronavirus disease 2019 (COVID-19), may cause hyperglycemia and additional systemic complexity in metabolic parameters. It is unsure even if the virus itself causes type 1 or type 2 diabetes mellitus (T1DM or T2DM). Furthermore, it is still unclear whether even recuperating COVID-19 individuals have an increased chance to develop new-onset diabetes.

**Methods:**

We wanted to determine the impact of COVID-19 on the levels of adipokines, pancreatic hormones, incretins and cytokines in acute COVID-19, convalescent COVID-19 and control children through an observational study. We performed a multiplex immune assay analysis and compared the plasma levels of adipocytokines, pancreatic hormones, incretins and cytokines of children presenting with acute COVID-19 infection and convalescent COVID-19.

**Results:**

Acute COVID-19 children had significantly elevated levels of adipsin, leptin, insulin, C-peptide, glucagon and ghrelin in comparison to convalescent COVID-19 and controls. Similarly, convalescent COVID-19 children had elevated levels of adipsin, leptin, insulin, C-peptide, glucagon, ghrelin and Glucagon-like peptide-1 (GLP-1) in comparison to control children. On the other hand, acute COVID-19 children had significantly decreased levels of adiponectin and Gastric Inhibitory Peptide (GIP) in comparison to convalescent COVID-19 and controls. Similarly, convalescent COVID-19 children had decreased levels of adiponectin and GIP in comparison to control children. Acute COVID-19 children had significantly elevated levels of cytokines, (Interferon (IFN)) IFNγ, Interleukins (IL)-2, TNFα, IL-1α, IL-1β, IFNα, IFNβ, IL-6, IL-12, IL-17A and Granulocyte-Colony Stimulating Factors (G-CSF) in comparison to convalescent COVID-19 and controls. Convalescent COVID-19 children had elevated levels of IFNγ, IL-2, TNFα, IL-1α, IL-1β, IFNα, IFNβ, IL-6, IL-12, IL-17A and G-CSF in comparison to control children. Additionally, Principal component Analysis (PCA) analysis distinguishes acute COVID-19 from convalescent COVID-19 and controls. The adipokines exhibited a significant correlation with the levels of pro-inflammatory cytokines.

**Conclusion:**

Children with acute COVID-19 show significant glycometabolic impairment and exaggerated cytokine responses, which is different from convalescent COVID-19 infection and controls.

**Supplementary Information:**

The online version contains supplementary material available at 10.1186/s12887-023-03971-w.

## Background

Severe Acute Respiratory Syndrome Coronavirus-2 (SARS-CoV-2), the causative agent for Coronavirus disease 2019 (COVID-19), was thought to be an exclusively respiratory virus [1]. However, growing experimental and clinical data implies that SARS-CoV-2 shows extrapulmonary manifestations, interrelated with diabetic complications, decreased renal function, cardiac dysfunction, and neurological, and gastrointestinal symptoms [2–9]. Moreover, SARS-CoV-2 may trigger a cytokine storm, an augmented immune response that creates a systemic proinflammatory environment, that might have a role in enabling insulin resistance and beta-cell hyperstimulation, finally resulting in beta-cell dysfunction and death [10]. Deregulation of metabolic function has been previously noticed in COVID-19 patients like hyperglycemia in type 2 diabetes mellitus (T2DM) individuals [11], ketoacidosis in diabetic and non-diabetic patients [12, 13] who had SARS-CoV-2 infection, and new-onset type 1 diabetes mellitus (T1DM) in the absence of autoantibodies [14–16]. Exaggerated pro-inflammatory response and dysregulated host response results in tissue damage [17–20]. The imbalance among pro and anti-inflammatory adipokines produced by adipose tissue contributes to the development of insulin resistance and type II diabetes, atherosclerosis, and non-alcohol fatty liver disease (NAFLD) [21]. A multicentric study showed an 80% upsurge in newly diagnosed T1DM in children amid the COVID-19 pandemic [22]. Destruction of pancreatic β-cell function and apoptosis may occur due to elevated levels of cytokines that might be induced by factors connected to the SARS-CoV-2 infection [3, 23], thus leading to impaired insulin secretion and ketosis [3]. It is unclear how SARS-CoV-2 could promote β-cell damage. It might happen through immune-mediated β-cell destruction or alteration in the function of β-cells, which results in infection-related diabetes consistent with the present World Health Organization (WHO) categorization [24]. However, it is unclear whether COVID-19 has any impact on hyperglycemia. An earlier report described the consequences of SARS-CoV-2 on the pancreas [25]. Similarly, one other study observed that SARS-CoV-2 impaired pancreatic function, suggesting that SARS-CoV-2 may result in acute insulin-dependent diabetes mellitus [26]. Hyperglycemia with or without a history of DM is a powerful prognosticator of hospitalised consequences [27]. Individuals with hyperglycemia have sevenfold increased mortality compared with those who had controlled blood glucose levels [27, 28]. Even though several reports indicate diabetes as a key parameter for COVID-19, it is still uncertain what impact COVID-19 has on cytokines and glyco -metabolic parameters, including inflammatory cytokines, adipokines, pancreatic hormones and incretins and pathological mechanisms. In light of the aforementioned studies, we wanted to investigate the serum concentrations of inflammatory cytokines, adipokines, pancreatic hormones and incretins in acute COVID-19, convalescent COVID-19 and control children. These analyses were implemented to evaluate alterations of adipokine and cytokines levels among COVID-19 patients in comparison to patients without SARS-CoV-2 infection. In addition, we elucidated the relationship between inflammatory cytokines, adipokines, pancreatic hormones and incretins serum levels and COVID-19 severity.

## Methods

### Study design and participants

We carried out an observational study and performed a comprehensive immunological analysis on plasma samples from children with confirmed SARS-COV-2 infection presenting to Sri Ramachandra Institute of Higher Education and Research (SRIHER), a tertiary hospital in Chennai, India from Aug 2020 to Aug 2021. Above and under the age of 16 years informed consent was obtained from both the adult participants and the parent(s)/guardian(s) of all under-16s. Both children with acute infection (RT-PCR positive) and previous infection (RT-PCR negative but IgG positive) were included. Previous infections are hereafter termed convalescent COVID-19. Control samples were healthy children who had presented to Kanchi Kamakoti CHILDS Trust Hospital, for elective surgery and were both RT-PCR and IgG negative. These samples were from another paediatric COVID-19 study [29]. Blood samples were collected in EDTA tubes (BD Biosciences), and plasma samples were collected through centrifugation and stored at –80 °C till transportaction to the National Institute for Research in Tuberculosis (NIRT), Chennai for serological and immunological analysis. All methods were carried out in accordance with all the institutional committee guidelines and regulations. Multiplex assays on all samples were carried out at the same time to restrain the batch effect.

### Clinical data

The SRIHER study team approached caregivers of children with SARS-CoV-2 infection during the above-mentioned period for participation in the study. Demographic, epidemiological, clinical and laboratory data were collected on a standardised case report form. Acute COVID-19 disease and the severity of the disease were defined according to the Ministry of Health and Family Welfare (MOHFW) guidelines (Guidance document on appropriate management of suspect or confirmed cases of COVID-19, https://www.mohfw.gov.in. 2021) issued by the Government of India.

### SARS-CoV-2 RT-PCR test

SARS-CoV-2 real-time reverse-transcriptase polymerase chain reaction (RT-PCR) was performed by Indian Council of Medical Research (ICMR) approved laboratories. Results of RT-PCR were shared along with the clinical data by the SRIHER study team.

### SARS-CoV-2 antibody assay

Serological and immunological assays were performed at the National Institutes of Health -National Institute for Research in Tuberculosis—International Center for Excellence in Research laboratory (NIH-NIRT-ICER), NIRT, Chennai. Antibodies were quantified in plasma using iFlash© SARS-CoV-2 IgG chemiluminescence antibody assay (CLIA) (YHLO Biotechnology Corporation, Shenzhen, China) according to the manufacturer’s instructions. An antibody titre of > 10 AU/ml was considered positive.

### Measurement of plasma adipocytokines levels

Plasma levels of pancreatic hormones (C-peptide, insulin and glucagon), adipocytokines (adiponectin, adipsin, resistin, leptin, Visfatin, Plasminogen Activator Inhibitor-1 (PAI-1)) and the levels of Incretins (Ghrelin, Gastric Inhibitory Peptide (GIP) and Glucagon-like peptide-1 (GLP-1)) were measured using a Bioplex multiplex assay system (Bio-Rad, Hercules, CA).

### Multiplex assays

Circulating plasma levels of cytokines were measured by the Luminex Magpix Multiplex Assay system (Bio-Rad, Hercules, CA) using the Luminex Human Magnetic Assay kit (R & D systems). The lowest detection limits for cytokines were as follows: IFNγ, 5.7 pg/mL; IL-2, 3.6 pg/mL; TNFα, 12.4 pg/mL; IL-1α, 10.6 pg/mL; IL-1β, 3.5 pg/mL; IFNα, 3.9 pg/mL; IFNβ 3.25 pg/mL; IL- 6, 9.0 pg/mL; IL-12, 18.5 pg/mL; IL-17A, 9 pg/mL; G-CSF, 8.4 pg/mL and IL-10, 32.2 pg/mL.

### Statistical analysis

Children were categorised into three groups acute COVID-19 (SARS-COV-2, RT-PCR positive), Convalescent-COVID-19 (SARS-CoV-2, IgG positive) and Control (both serology and RT-PCR negative). The Shapiro-Wilks test was used to assess the normality of the data. Continuous variables are presented as median (ranges), and categorical variables are reported as numbers and proportions. Power calculations were conducted to obtain a power of 80% and an α error of 0.05%. Statistically significant differences between acute COVID-19, convalescent COVID-19 children and controls were analysed using the Kruskal–Wallis test with Dunn’s multiple comparisons. *P* < 0.05 was considered statistically significant. Analyses were performed using Graph-Pad PRISM Version 9.0 (GraphPad Software, CA, USA). Principal component analysis (PCA) and Hierarchical analysis were done using JMP 16.0 (SAS, Cary, NC, USA) statistical software.

## Results

### Basic characteristics

We carried out the immunological assays on plasma samples of 51 children. Of these 51 children, 12 had acute COVID-19 (RT-PCR positive, IgG negative) and 19 children had recovered from acute infection (convalescent; IgG positive). Blood sampling in the acute infection group was done, prior to giving any medications, on days 10 to 12 of symptoms and in convalescent children, it was performed 3 months after acute infection. The median duration of blood sampling in convalescent children was 12 weeks since proven COVID-19 illness. Children presenting with acute COVID-19 had mild to moderate symptoms and did not require any intensive care support. Of the 12 acute COVID-19 children, 1 (8%) had a fever, 1 (8%) had a sore throat, 2 (17%) had a loss of smell and 1 (8%) had fatigue. Six of these children were admitted to the paediatric ward for 24 h for observation. All children received supportive care with antipyretics and intravenous fluids. None of the children received immunomodulatory therapy. Among the 19 convalescent children, 7 (37%) had a fever, 1 (5%) presented with a fever, 1 (8%) had a sore throat, 2 (10%) had respiratory symptoms, 1 (5%) had a loss of smell and 1 (5%) had gastrointestinal symptoms, during acute infection phase. However, none of the children required hospitalisation during acute illness. For comparison, we included 20 healthy children from another study [29] who had presented for elective surgery and were both RT-PCR and IgG negative. Further demographics and clinical presentation of participant children are described in Tables 1 and 2.

**Table 1:** Demographics of the study population

Parameter	Acute COVID-19	Convalescent COVID-19	Controls	*P* value
	*n* = 12	*n* = 19	*n* = 20	
	Median (range)			
Age	7 (2 months–16 yrs)	8 (7 months–17 yrs)	8 (1.2–15.6 yrs)	0.7452
Male	5 (42%)	11 (58%)	14 (70%)	
Female	7 (58%)	8 (42%)	6 (30%)	
Weight (Kg)	22 (4.2–60)	21.5 (9.5–69)	23 (8–65)	0.6894
Height (cm)	123 (58–165)	123 (73–174)	127 (73–181)	0.7163
BMI (kg/m^2^)	18.7 (12.4–23.6)	18 (13.1–26.9)	19 (13.2–27.2)	0.8683
Random Blood Glucose (RBG) mg/dL	119 (98–128)	110 (95–122)	102 (96–120)	0.6438

**Table 2:** Clinical COVID-19 symptoms of the study group

Parameter	Acute COVID-19	Convalescent COVID-19	*P* value
	*n* = 12	*n* = 19	
	COVID-19 Symptoms		
Fever	1 (8%)	7 (37%)	0.0001
Sore Throat	1 (8%)	1 (5%)	0.0001
Respiratory Symptoms	0	2 (10%)	NA
Loss of smell and taste	2 (17%)	1 (5%)	0.0001
Gastro intestinal symptoms	0	1 (5%)	NA
Abdominal pain	0	0	NA
Nausea	0	0	NA
Rash	0	0	NA
Hypertension / Shock	0	0	NA
Fatigue	1 (8%)	0	NA
Myalgia	0	0	NA
Pneumonia	0	0	NA
Mucocutaneous inflammation	0	0	NA
Underlying conditions	0	0	NA

### Acute COVID-19 children associated with altered levels of adipocytokines

**Fig. 1:**
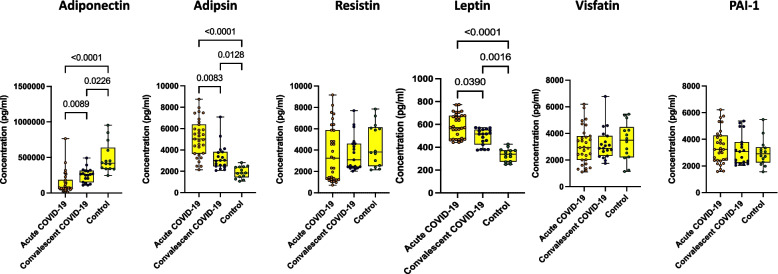
Acute COVID-19 children are associated with altered levels of adipocytokines. The plasma levels of adipocytokines such as Adiponectin, Adipsin, Resistin, Leptin, Visfatin and PAI-1 were measured in acute COVID-19 (*n* = 12), Convalescent COVID-19 (*n* = 19) and control children (*n* = 20). The data are represented as Box and whiskers with each circle representing a single individual. *P* values were computed using the Kruskal–Wallis test with Dunn’s post-hoc for multiple comparisons

We wanted to determine the impact of SARS-COV-2 on adipocytokines levels in children with various clinical spectrums. We measured the adipocytokines levels (Adipokines, Adipsin, Resistin, Leptin, Visfatin and Plasminogen Activator Inhibitor-1 (PAI-1)) in acute, convalescent COVID-19 and control children. As depicted in Fig. 1, acute COVID-19 children exhibited lower levels of Adiponectin in comparison to convalescent and control children. Similarly, convalescent COVID-19 children also exhibited lower levels of Adiponectin in comparison to control children. On the other hand, acute COVID-19 children exhibited higher levels of Adipsin and Leptin in comparison to convalescent and control children. Similarly, convalescent COVID-19 children also exhibited higher levels of Adipsin and Leptin in comparison to control children. Other adipocytokines such as Resistin, Visfatin and PAI-1 did not exhibit statistically significant differences among the acute, convalescent and control groups of children. Geomean values are shown in S.Table.1. Our results suggest that SARS-CoV-2 influences adipocytokines. Both acute COVID-19 and convalescent COVID-19 children are characterized by distinct plasma levels of adipocytokines.

### Acute COVID-19 children associated with elevated levels of pancreatic hormones

**Fig. 2:**
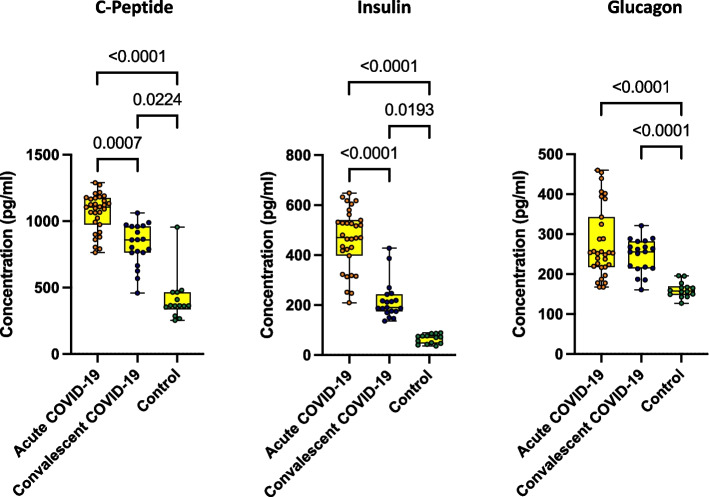
Acute COVID-19 children are associated with elevated levels of pancreatic hormones. The plasma levels of pancreatic hormones such as C-peptide, Insulin and glucagon were measured in acute COVID-19 (*n* = 12), Convalescent COVID-19 (*n* = 19) and control children (*n* = 20). The data are represented as Box and whiskers with each circle representing a single individual. *P* values were computed using the Kruskal–Wallis test with Dunn’s post-hoc for multiple comparisons

Next, we wanted to examine the impact of SARS-COV-2 on pancreatic hormone levels in children with various clinical spectrum of COVID-19. We measured the pancreatic hormone levels (C-peptide, Insulin and Glucagon) in acute, convalescent COVID-19 and control children. As depicted in Fig. 2, acute COVID-19 children exhibited elevated levels of C-peptide, Insulin and Glucagon in comparison to convalescent and control children. Similarly, convalescent COVID-19 children also exhibited elevated levels of C-peptide, Insulin and Glucagon in comparison to control children. Geomean values are shown in S.Table.1.

### Acute COVID-19 children associated with altered levels of Incretins

**Fig. 3:**
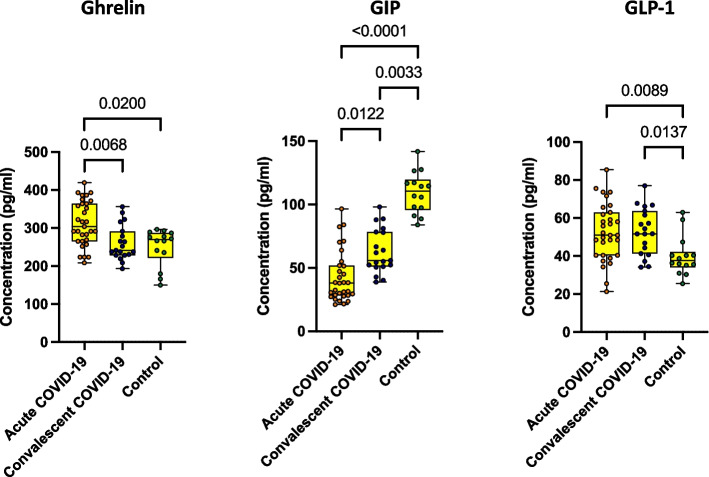
Acute COVID-19 children are associated with altered levels of Incretins. The plasma levels of Incretins such as Ghrelin, GIP and GLP-1 were measured in acute COVID-19 (*n* = 12), Convalescent COVID-19 (*n* = 19) and control children (*n* = 20). The data are represented as Box and whiskers with each circle representing a single individual. *P* values were computed using the Kruskal–Wallis test with Dunn’s post-hoc for multiple comparisons

Subsequently, we wanted to examine the impact of SARS-CoV-2 on the levels of Incretins in children with different clinical spectrums of COVID-19. We measured the levels of incretins (Ghrelin, Gastric Inhibitory Peptide (GIP) and glucagon-like peptide-1 (GLP-1) in acute, convalescent COVID-19 and control children. As depicted in Fig. 3, acute COVID-19 children exhibited increased levels of Ghrelin in comparison to convalescent and control children. Acute COVID-19 children exhibited lower levels of GIP in comparison to convalescent and control children. Similarly, convalescent COVID-19 children also exhibited lower levels of GIP in comparison to control children. On the other hand, acute COVID-19 children exhibited higher levels of GIP-1 in comparison to control children. Similarly, convalescent COVID-19 children also exhibited higher levels of GIP-1 in comparison to control children. Ghrelin did not exhibit statistically significant differences among the acute, convalescent and control groups of children. Geomean values are shown in S.Table.1.

### Acute COVID-19 children associated with altered levels of cytokines

**Fig. 4:**
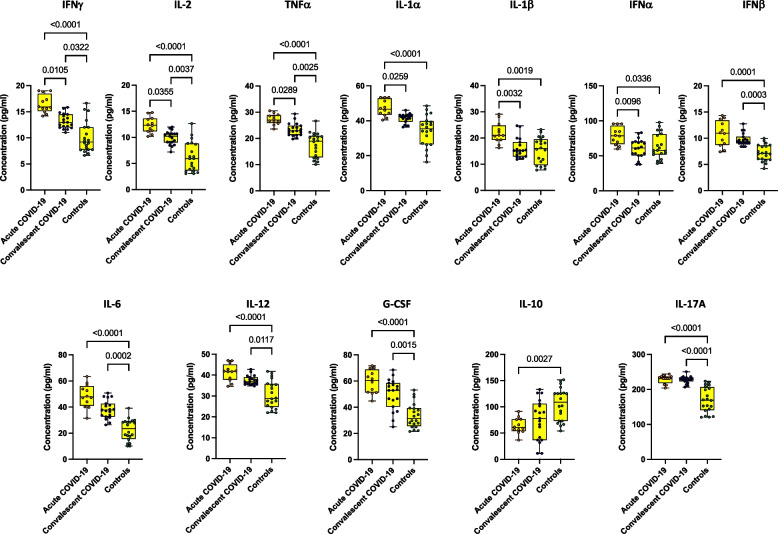
Acute COVID-19 children are associated with altered levels of cytokines. The plasma levels of IFNγ, IL-2, TNFα, IL-1α, IL-1β, IFNα, IFNβ, IL-6, IL-12, IL-17A and G-CSF were measured in acute COVID-19 (*n* = 12), Convalescent COVID-19 (*n* = 19) and control children (*n* = 20). The data are represented as Box and whiskers with each circle representing a single individual. *P* values were computed using the Kruskal–Wallis test with Dunn’s post-hoc for multiple comparisons

To determine the systemic cytokine responses among various clinical phenotypes, we quantified the plasma levels of Type 1, (Interleukins-IL) IL-1α, IL-1β, Type 1 Interferons- IFN) IFNs and other pro-inflammatory cytokines (Fig. 4). Acute COVID-19 children had significantly elevated levels of cytokines, IFNγ, IL-2, TNFα, IL-1α, IL-1β and IFNα in comparison to convalescent children. Also, acute COVID-19 children had significantly elevated levels of cytokines, IFNγ, IL-2, TNFα, IFNβ, IL-6, IL-12, IL-17A and Granulocyte-Colony Stimulating Factors G-CSF in comparison to control children. Convalescent COVID-19 children had elevated levels of IFNγ, IL-2, TNFα, IL-1α, IL-1β, IFNα, IFNβ, IL-6, IL-12, IL-17A, IL-10 and G-CSF in comparison to control children. Thus, acute COVID-19 in children is characterized by elevated plasma levels of cytokines in comparison to convalescent COVID-19 and control children.

### Plasma diabetic-related parameters and cytokines can strongly discriminate acute COVID-19 from convalescent COVID-19 and control children

**Fig. 5:**
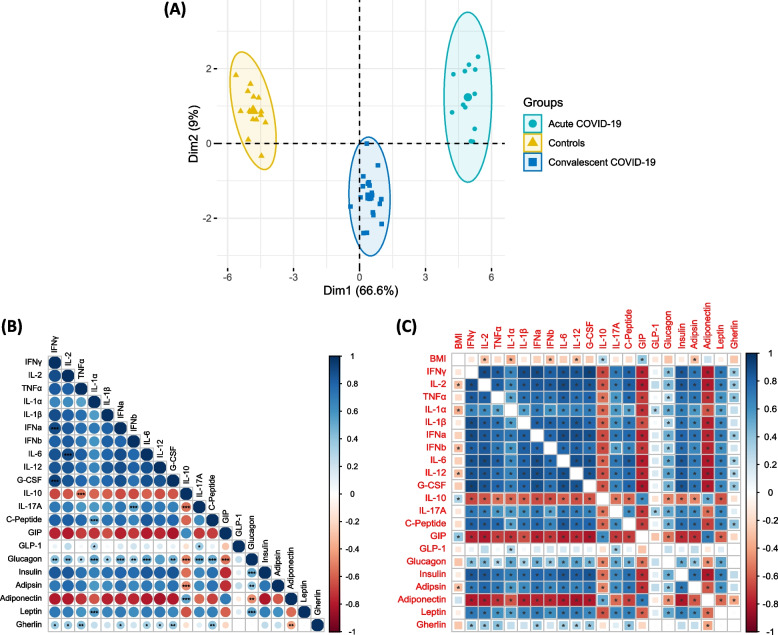
Plasma diabetic parameters can strongly discriminate acute COVID-19 from convalescent COVID-19 and control children. **A** PCA (Principal component analysis) plot computing normalized cellular subsets after excluding those factors with commonalities as low as 0.5. We used C-Peptide, GIP, GLP-1, Glucagon, Insulin, Adipsin and Adiponectin parameters in a combination of three different experimental groups acute COVID-19 (Coloured in green) vs convalescent COVID-19 (Coloured in blue) vs controls (Coloured in yellow). **B** (A). Multiparametric matrix correlation plot of Adiponectin, Adipsin, Resistin, Leptin, Visfatin and PAI-1, C-peptide, Insulin and glucagon, Ghrelin, GIP and GLP-1 and IFNγ, IL-2, TNFα, IL-1α, IL-1β, IFNα, IFNβ, IL-6, IL-12, IL-17A and G-CSF levels in all children with acute COVID-19, Convalescent COVID-19 and control children Spearman’s correlation method was used for the analysis. **C** Multiparametric matrix correlation plot of BMI with Adiponectin, Adipsin, Resistin, Leptin, Visfatin and PAI-1, C-peptide, Insulin and glucagon, Ghrelin, GIP and GLP-1 and IFNγ, IL-2, TNFα, IL-1α, IL-1β, IFNα, IFNβ, IL-6, IL-12, IL-17A and G-CSF levels in all children with acute COVID-19, Convalescent COVID-19 and control children Spearman’s correlation method was used for the analysis.* indicates *p* = 0.05, ** indicates *p* = 0.01 and *** indicates *p* = 0.001, the blue colour denotes the positive correlation and the red colour denotes the negative correlation

Further, we wanted to determine the potential of plasma diabetic parameters in differentiating acute COVID-19 from convalescent COVID-19 and control children. We performed PCA (principal component analysis) and as plotted in Fig. 5A, Our PCA data demonstrates the ability of these markers to differentiate acute COVID-19, convalescent COVID and control children. We observed that PC1 variation is 66.6% and PC2 variation is about 9% of the variability by adding normalized diabetic and cytokine parameters after omitting those factors with commonalities as low as 0.5. in children with acute COVID-19, convalescent COVID-19 and control children. Thus, diabetic parameters and cytokines strongly differentiate acute COVID-19 from convalescent and control children.

### Correlation of plasma diabetic-related parameters and cytokines

Next, we wanted to know the relationship between plasma diabetic-related parameters and cytokines. Therefore, we performed the Spearman rank correlation analysis to determine the association between plasma diabetic-related parameters with cytokines IFNγ, IL-2, TNFα, IL-1α, IL-1β, IFNα, IFNβ, IL-6, IL-12, IL-17A, IL-10 and G-CSF. As shown in Fig. 5B. plasma levels of Glucagon positively correlated with IFNγ, IL-2, TNFα, IL-1α, IL-1β, IFNα, IFNβ, IL-6, IL-17A and G-CSF. C-Peptide and Leptin exhibited a positive correlation with IL-1α. Ghrelin exhibited a positive correlation with IFNγ, IL-2, TNFα, IFNα, IL-6, IL-12, and G-CSF. GLP-1 exhibited a weak positive correlation with IL-1α and IL-17A. In contrast, Glucagon and Adipsin correlated negatively with IL-10. Further, we performed the correlation analysis between BMI Vs glycemic parameters and cytokines as shown in Fig. 5C. BMI exhibited a significant negative correlation with Adipsin, IL-2, IL-1α, IFNβ, IL-12, IL-10 and G-CSF. In contrast, BMI exhibited a positive correlation with GIP.

### Multivariate regression analysis of cytokine, glycemic parameters interaction

The influence of confounding variables on children with acute and convalescent COVID-19 with different analytes was evaluated in this study using multivariate regression analysis. As illustrated in Table 3, even after adjusting for the influence of age and sex, the levels of biochemical parameters such as RBG, BMI, diabetic parameters such as insulin, C-peptide, GIP, adiponectin, adipsin, resistin, ghrelin, leptin, visfatin and PAI-1; cytokines like IFN-γ, IL-2, TNF-α, IL-10, IL-1α, IFNα, IFNβ, IL-6 and IL-12 were all significantly influenced by COVID-19 disease. Therefore, our data corroborate that COVID-19 disease has a great impact on numerous significant factors in glycemic and cytokines parameters, including blood glucose levels, the levels of adipocytokines and the more conventional cytokines.

**Table 3:** Multiple logistic regression analysis

**Factors**	**CRUDE OR (95% CI)**	***P* Value**	**Adjusted OR (95% CI)**	***P* Value**
Age^a^	1.23 (0.61–3.33)	0.542		
Gender^a^				
Female	1.00			
Male	0.79 (0.47–1.65)	0.475		
RBG (mg/dl)	1.019 (1.003–1.034)	0.015	1.013 (1.001–1.024)	0.029
BMI Kg/m^2^	0.01 (0.01–0.02)	< 0.001	0.01 (0.01–0.02)	< 0.001
Insulin (pg/ml)	0.243 (0.153–0.513)	< 0.001	0.227 (0.183–0.573)	< 0.001
Leptin (pg/ml)	2.176 (1.391–4.586)	< 0.001	2.108 (1.259–4.464)	0.001
Adiponectin (pg/ml)	1.527 (1.496–2.492)	< 0.001	1.308 (1.489–2.454)	< 0.001
Adipsin (pg/ml)	2.345 (1.368–4.032)	< 0.001	2.237 (1.366–4.011)	< 0.001
C-peptide (pg/ml)	1.116 (1.002–1.023)	0.004	1.129 (1.011–1.045)	0.004
Glucagon (pg/ml)	0.899 (0.987–1.000)	0.305	0.899 (0.985–1.000)	0.194
Ghrelin (pg/ml)	1.001 (1.000–1.003)	0.018	1.003 (1.000–1.012)	0.039
GLP-1 (pg/ml)	1.000 (0.969–1.011)	0.394	0.999 (0.968–1.011)	0.737
GIP (pg/ml)	1.000 (0.967–1.001)	0.022	1.000 (0.969–1.001)	0.024
IFNγ (pg/ml)	1.001(1.000–1.004)	0.002	1.001 (1.000–1.004)	≤ 0.001
IL-2 (pg/ml)	0.993 (0.986–0.992)	0.001	0.974 (0.963–0.992)	0.008
TNF-α (pg/ml)	0.992 (0.996–0.981)	0.003	0.984 (0.979–0.981)	0.052
IL-17A (pg/ml)	0.994 (0.986–0.994)	0.004	0.995 (0.982–1.001)	0.309
IL-1α (pg/ml)	0.997 (0.958–0.989)	0.011	0.981 (0.961–0.959)	0.025
IL-1β (pg/ml)	1.005 (0.976–1.015)	0.044	1.010 (0.998–1.021)	0.307
IFN-α (pg/ml)	0.994 (0.993–0.996)	0.009	0.983 (0.975–0.992)	0.021
IFN-β (pg/ml)	1.011 (1.002–1.023)	0.008	1.021 (1.011–1.045)	0.031
IL-6 (pg/ml)	0.984 (0.979–0.989)	0.009	0.995 (0.963–0.989)	0.028
IL-12 (pg/ml)	0.996 (0.913–1.010)	0.119	0.883 (0.764–0.958)	0.089
G-CSF (pg/ml)	0.996 (0.993–1.003)	0.305	0.996 (0.993–1.003)	0.190
IL-10 (pg/ml)	1.040 (1.006–1.063)	0.026	1.061 (0.998–1.060)	0.185

^a^Adjusted for age and gender

## Discussion

In this study, we describe the comprehensive immune metabolic parameters (adipokines, pancreatic hormones and incretins) and cytokines of children with acute and convalescent COVID-19 and found that acute COVID-19 children exhibit altered levels of adipokines, pancreatic hormones and incretins, which discriminates them from convalescent COVID-19 and control children. Dysfunction of adipokine production is linked with metabolic disorders like diabetes mellitus, obesity and metabolic syndrome [30]. SARS-CoV-2 infection induces protective responses in the form of the combined immune response of primary cytokine secretion and stimulation of antiviral IFN response following immune-cell recruitment [31]. In other viral infections, the effect of metabolic modifications has been reported [32, 33] such as influenza A (H1N1) influenza or the Middle East respiratory syndrome-related coronavirus (MERS-CoV) [34, 35]. Similarly, recent data indicate that reciprocal interaction between COVID-19 and diabetes consists of a perplexing pathophysiological component elemental for hyperglycemia and overall hypometabolic disorder [10, 36]. SARS-COV-2 is associated with new-onset diabetes manifesting as acute hyperglycemia or diabetic ketoacidosis in post-COVID-19 and/or acute COVID-19 patients without diabetes history or with existing diabetes, either during the course of the disease or after convalescence [37]. Consistent with adult studies, we found that acute COVID-19 is related to altered glucoregulatory hormones in the paediatric population. Acute COVID-19 children exhibited decreased adiponectin, and GIP and increased adipsin, leptin, C-peptide, insulin and ghrelin when compared with convalescent and control group of children without prior history of diabetes.

A recent study on adult COVID-19 with ARDS patients showed decreased adiponectin compared to the Intensive Care Unit (ICU) control [38]. Adipocytes secrete several pro-inflammatory mediators and among them, TNF-α has been proposed to develop a link between IR, obesity and T2DM [39]. Several adipocytokines, like adiponectin, Resistin, visfatin, and leptin are secreted by Adipocytes. These parameters are considered to be related to insulin resistance, and inflammatory complications [40, 41]. T2DM and obese individuals are shown to be associated with decreased levels of adiponectin which has anti-diabetic, anti-atherogenic, and anti-inflammatory properties [38]. In a recent adult study from Italy, normal glycemic individuals had insulin resistance and elevated cytokine levels in hospitalized COVID-19 individuals [10, 42]. We found that acute COVID-19 children were associated with lower levels of adiponectin than convalescent and control children indicating that lower levels of adiponectin related to COVID-19. Adipsin, another adipokine, has a function in the support of beta-cell survival and insulin production [38, 43, 44]. In adults, adipocyte deregulation associated with leptin might be promoting the progression of ARDS in COVID-19 patients [45]. Leptin provokes an inflammatory phenotype, suggesting that an increased level of leptin in obesity could increase the chances of pulmonary inflammation in COVID-19 infection [45]. Leptin is involved in the endocrine signalling mechanism and cytokine-mediated alteration in the body which could play a significant role in the progression of severe COVID-19 infection [45, 46]. We observed that acute COVID-19 children exhibited higher concentrations of adipsin, leptin, C-peptide, ghrelin and GIP in comparison to convalescent and control children, whereas the adiponectin concentrations were significantly decreased in acute COVID-19 in comparison to convalescent and control children. In contrast, glucagon and GLP-1 were found to be higher in acute COVID-19 than in the control children. These findings suggest that COVID-19 could induce adipocyte dysfunction [38]. Adipose tissue may completely establish a viral repository of SARS-CoV-2, and worsen the severity of COVID-19 via augmentation of the immune and cytokine provocations [47]. A recent case report study observed that SARS-CoV-2 patients with new-onset Diabetic Ketoacidosis (DKA) exhibited the opposite for T1DM autoantibody, signifying the chance of pancreatic beta-cell impairment or damage due to COVID-19 [16]. The possible mechanism for these altered levels of adipocytokines, pancreatic hormones and incretins might be due to the virus could be attaching to angiotensin-converting enzyme 2 (ACE2) receptors in pancreatic beta cells, leading to pleiotropic changes in glucose metabolism [48, 49].

Studies have shown that decreased levels of adiponectin and increased levels of inflammatory cytokines like IL-6, and TNFα contribute directly to insulin resistance [50] and also directly prevent adiponectin secretion from adipocytes [30]. Cordeiro A et al. reported that chronic inflammation induces excessive levels of cytokines like IL-6, TNF-α, and IL-1β, together with diminished levels of anti-inflammatory cytokines IL-10 [51]. Elevated levels of TNF-α and IL-6 in cytokine storm damage the function of pancreatic β-cell and restrain the production of insulin. Both Insulin Resistance and deficiency in the function of pancreatic β-cell could lead to the development and progression of hyperglycemia in COVID-19 patients [52]. Various studies determined that IL-6 and TNFα could promote insulin resistance [30]. Exaggerated inflammation with a deregulatory immune response induces a cytokine storm associated with the pathogenesis of COVID-19 [53]. We observed a positive link between the adipocytokines and the pro-inflammatory cytokines. In addition, our data on PCA revealed distinct discrimination of cytokines and chemokines expressing patterns of acute and convalescent COVID-19 clinical spectrum and control group of children.

Our study has some limitations. First, we analysed metabolic parameters (adipokines, pancreatic hormones, incretins and cytokines) in very small numbers from a single centre, implying further research is warranted to provide definitive answers. Second, we did not collect the diabetic history in the family, which is important as the genetic predisposition of the patients may play a role in immune responses. Despite the limitations, our data show that adipose tissue dysfunction and exaggerated cytokine responses are features of COVID-19 in children. Systemic inflammation in acute COVID-19 may also contribute to adipose dysfunction. We also, observed that acute COVID-19 children exhibit elevated levels of inflammatory cytokines and provide biosignatures which discriminate acute COVID-19 children from convalescent COVID-19 and control children.

These observations warrant future studies with larger cohorts of children for in-depth knowledge of the impact of SARS-CoV-2 infection on adipokines, pancreatic hormones incretins and cytokines. Moreover, such a strategy could be of help to develop specific adipokine-targeted treatment strategies that can be implemented in the acute setting. It is important to understand whether SARS-COV-2 infection may be a factor related to the new diagnosis of DM in children. Understanding the biological impairment caused by SARS-COV-2 may provide in-depth knowledge of the pathophysiology of the disease and eventually lead to the advancement of proposed adjuvant therapies in addition to antiviral drugs and prevent the onset of diabetes in children.

## Supplementary Information

**Additional file 1: Supplementary Table 1.** Geo mean of.

## Data Availability

All data generated or analysed during this study are included in this published article [and its supplementary information files].
