# Supplementary material for: Alterations of adipokines, pancreatic hormones and incretins in acute and convalescent COVID-19 children
Source: BMC Pediatr. 2023 Apr 3;23:156. doi: 10.1186/s12887-023-03971-w (PMC10068212; doi:10.1186/s12887-023-03971-w)
Supplement: Supplementary file 1 — Additional file 1: Supplementary Table 1. Geo mean of. [file 12887_2023_3971_MOESM1_ESM.docx]

**Supplementary Table.1. Geo mean of**

| **Parameters** | **Acute COVID-19** | **Convalescent**  **COVID-19** | **Control** | **p value** |
| --- | --- | --- | --- | --- |
| **Adiponectin** | 92919 | 230285 | 457568 | <0.0001 |
| **Adipsin** | 4807 | 3149 | 1833 | <0.0001 |
| **Resistin** | 2774 | 3415 | 4084 | 0.2995 |
| **Leptin** | 582 | 485.6 | 329.4 | <0.0001 |
| **Visfatin** | 2770 | 3032 | 3061 | 0.7402 |
| **PAI-1** | 3187 | 3050 | 2872 | 0.6199 |
| **C-Peptide** | 1056 | 805.9 | 388.9 | <0.0001 |
| **Insulin** | 446.4 | 208.2 | 62.07 | <0.0001 |
| **Glucagon** | 265.5 | 243.2 | 159.9 | <0.0001 |
| **Ghrelin** | 306.8 | 256.9 | 244.4 | 0.002 |
| **GIP** | 38.94 | 60.83 | 108.2 | <0.0001 |
| **GLP-1** | 50.49 | 51.49 | 38.77 | 0.0056 |
| **IFNγ** | 16.41 | 13.11 | 9.783 | <0.0001 |
| **IL-2** | 12.30 | 9.846 | 5.687 | <0.0001 |
| **TNFα** | 27.21 | 23.19 | 16.99 | <0.0001 |
| **IL-1α** | 46.97 | 41.31 | 33.08 | <0.0001 |
| **IL-1β** | 21.57 | 15.71 | 14.39 | 0.0010 |
| **IFNα** | 77.87 | 59.02 | 61.26 | 0.0086 |
| **IFNβ** | 10.62 | 9.651 | 7.028 | <0.0001 |
| **IL-6** | 47.82 | 37.50 | 21.00 | <0.0001 |
| **IL-12** | 41.16 | 37.49 | 29.98 | <0.0001 |
| **G-CSF** | 58.84 | 48.33 | 32.46 | <0.0001 |
| **IL-10** | 62.22 | 61.78 | 100.7 | 0.0023 |
| **IL-17A** | 228.5 | 227.4 | 166.4 | <0.0001 |
